# Efficient and Directive Generation of Two Distinct Endoderm Lineages from Human ESCs and iPSCs by Differentiation Stage-Specific SOX17 Transduction

**DOI:** 10.1371/journal.pone.0021780

**Published:** 2011-07-07

**Authors:** Kazuo Takayama, Mitsuru Inamura, Kenji Kawabata, Katsuhisa Tashiro, Kazufumi Katayama, Fuminori Sakurai, Takao Hayakawa, Miho Kusuda Furue, Hiroyuki Mizuguchi

**Affiliations:** 1 Laboratory of Biochemistry and Molecular Biology, Graduate School of Pharmaceutical Sciences, Osaka University, Suita, Osaka, Japan; 2 Laboratory of Stem Cell Regulation, National Institute of Biomedical Innovation, Ibaraki, Osaka, Japan; 3 Laboratory of Biomedical Innovation, Graduate School of Pharmaceutical Sciences, Osaka University, Suita, Osaka, Japan; 4 Pharmaceutics and Medical Devices Agency, Chiyoda-ku, Tokyo, Japan; 5 Pharmaceutical Research and Technology Institute, Kinki University, Higashiosaka, Osaka, Japan; 6 JCRB Cell Bank, Division of Bioresources, National Institute of Biomedical Innovation, Ibaraki, Osaka, Japan; 7 Laboratory of Cell Processing, Institute for Frontier Medical Sciences, Kyoto University, Sakyo-ku, Kyoto, Japan; 8 The Center for Advanced Medical Engineering and Informatics, Osaka University, Suita, Osaka, Japan; VIB & Katholieke Universiteit Leuven, Belgium

## Abstract

The establishment of methods for directive differentiation from human embryonic stem cells (ESCs) and induced pluripotent stem cells (iPSCs) is important for regenerative medicine. Although Sry-related HMG box 17 (SOX17) overexpression in ESCs leads to differentiation of either extraembryonic or definitive endoderm cells, respectively, the mechanism of these distinct results remains unknown. Therefore, we utilized a transient adenovirus vector-mediated overexpression system to mimic the SOX17 expression pattern of embryogenesis. The number of alpha-fetoprotein-positive extraembryonic endoderm (ExEn) cells was increased by transient SOX17 transduction in human ESC- and iPSC-derived primitive endoderm cells. In contrast, the number of hematopoietically expressed homeobox (HEX)-positive definitive endoderm (DE) cells, which correspond to the anterior DE *in vivo*, was increased by transient adenovirus vector-mediated SOX17 expression in human ESC- and iPSC-derived mesendoderm cells. Moreover, hepatocyte-like cells were efficiently generated by sequential transduction of SOX17 and HEX. Our findings show that a stage-specific transduction of SOX17 in the primitive endoderm or mesendoderm promotes directive ExEn or DE differentiation by SOX17 transduction, respectively.

## Introduction

There are two distinct endoderm lineages in early embryogenesis, the extraembryonic endoderm (ExEn) and the definitive endoderm (DE). The first of these lineages, the ExEn plays crucial roles in mammalian development, although it does not contribute to the formation of body cells. In early embryogenesis, a part of the inner cell mass of the blastocyst differentiates into the primitive endoderm (PrE). The PrE differentiates into the ExEn that composes the parietal endoderm, which contributes to the primary yolk sac, and the visceral endoderm, which overlies the epiblast [1], [2]. In contrast, the second of the endoderm lineages, the DE arises from the primitive streak (PS), which is called the mesendoderm [3]. The DE has the ability to differentiate into the hepatic and pancreatic tissue [4].

The establishment of human embryonic stem cells (ESCs) [5] and human induced pluripotent stem cells (iPSCs) [6], [7] has opened up new opportunities for basic research and regenerative medicine. To exploit the potential of human ESCs and iPSCs, it is necessary to understand the mechanisms of their differentiation. Although growth factor-mediated ExEn or DE differentiation is widely performed, it leads to a heterogeneous population [8], [9], [10], [11]. Several studies have utilized not only growth factors but also modulation of transcription factors to control downstream signaling cascades [10], [12], [13]. Sox17, an Sry-related HMG box transcription factor, is required for development of both the ExEn and DE. In mice, during ExEn and DE development, Sox17 expression is first observed in the PrE and in the anterior PS, respectively [14]. Previous study showed that stable Sox17 overexpression promotes ExEn differentiation from mouse ESCs [12]. On the other hand, another previous study has demonstrated that DE progenitors can be established from human ESCs by stable expression of SOX17 [10]. The mechanism of these discrepancies which occurs in SOX17 transduction still remains unknown. Also, the role of SOX17 in human ExEn differentiation still remains unknown. Therefore, it is quite difficult to promote directive differentiation into either ExEn or DE cells by SOX17 transduction.

In this study, we utilized SOX17 as a stage-specific regulator of ExEn and DE differentiation from human ESCs and iPSCs. The human ESC- and iPSC-derived cells were transduced with SOX17-expressing adenovirus vector (Ad-SOX17), and the resulting phenotypes were assessed for their ability to differentiate into ExEn and DE cells *in vitro.* In addition, we examined whether SOX17-transduced cells have the ability to differentiate into the hepatic lineage. The results showed that stage-specific overexpression of the SOX17 transcription factor promotes directive differentiation into either ExEn or DE cells.

## Results

### The induction of human ESC-derived PrE cells and human ESC-derived mesendoderm cells

To determine the appropriate stage for SOX17 transduction, ExEn or DE cells were differentiated from human ESCs by a conventional method using BMP4 (20 ng/ml) or Activin A (100 ng/ml), respectively (Figures S1 and S2). Experiments for bidirectional differentiation using BMP4 and Activin A indicated that PrE cells were obtained on day 1 (Figure S1) and mesendoderm cells were obtained on day 3 (Figure S2). We expected that stage-specific SOX17 transduction into PrE cells or mesendoderm cells could promote ExEn or DE differentiation, because the time period of intiation of SOX17 expression was correlated with the time period of formation of PrE cells (day 1) (Figure S1C) and mesendoderm cells (day 3) (Figure S2C), respectively.

### PrE stage-specific SOX17 overexpression promotes directive ExEn differentiation from human ESCs

To examine the effect of forced and transient expression of SOX17 on the differentiation of human ESC- and iPSC-derived cells, we used a fiber-modified adenovirus (Ad) vector containing the EF-1α promoter and a stretch of lysine residues (KKKKKKK, K7) peptides in the C-terminal region of the fiber knob. The K7 peptide targets heparan sulfates on the cellular surface, and the fiber-modified Ad vector containing the K7 peptides has been shown to be efficient for transduction into many kinds of cells [15], [16].

**Figure 1:**
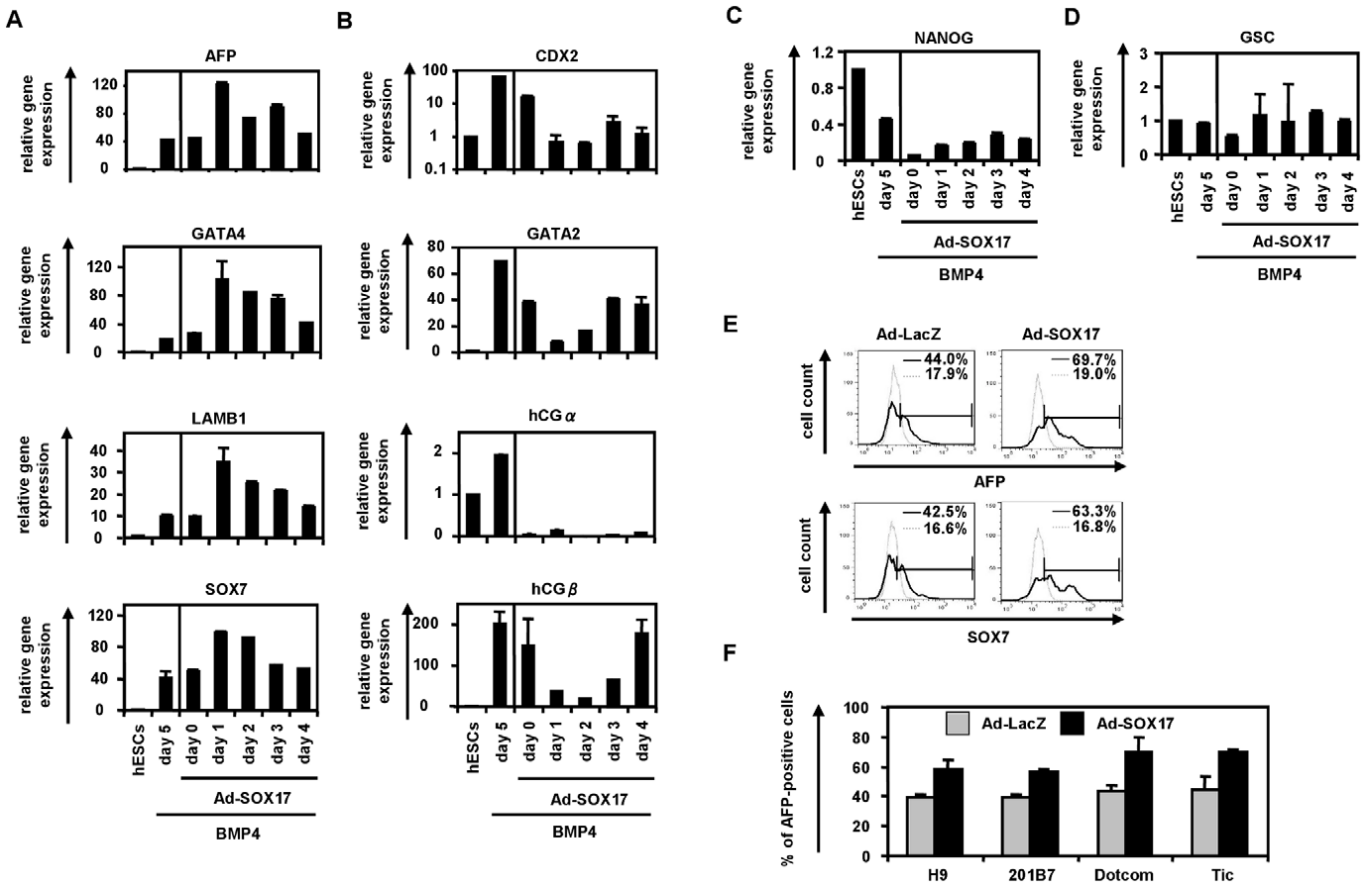
Efficient ExEn differentiation from human ESC- and iPSC-derived PrE cells by SOX17 transduction. (A–D) Undifferentiated human ESCs (H9) and BMP4-induced human ESC-derived cells, which were cultured with the medium containing BMP4 (20 ng/ml) for 0, 1, 2, 3, and 4 days, were transduced with 3,000 VP/cell of Ad-SOX17 for 1.5 h. Ad-SOX17-transduced cells were cultured with 20 ng/ml of BMP4, and then the gene expression levels of *(A)* the ExEn markers (AFP, GATA4, LAMB1, and SOX7), (B) the trophectoderm markers (CDX2, GATA2, hCGα, and hCGβ), (C) the pluripotent marker (NANOG), and (D) the DE marker (GSC) were examined by real-time RT-PCR on day 5 of differentiation. The horizontal axis represents the day on which the cells were transduced with Ad-SOX17. The expression levels of undifferentiated human ESCs on day 0 were defined 1.0. (E) On day 1, human ESC-derived PrE cells, which were cultured with the medium containing BMP4 for 1 day, were transduced with Ad-LacZ or Ad-SOX17 and cultured until day 5. The ExEn cells were subjected to immunostaining with anti-AFP or anti-SOX7 antibodies, and then analyzed by flow cytometry. (F) After Ad-LacZ or Ad-SOX17 transduction, the efficacies of ExEn differentiation from the human ES cell line (H9) and the three human iPS cell lines (201B7, Dotcom, and Tic) were compared on day 5 of differentiation. All data are represented as the means±SD (*n = *3).

Because the time period of initiation of SOX17 expression was correlated with the time period of formation of PrE cells (day 1) (Figure S1), we expected that stage-specific SOX17 transduction into PrE cells would promote ExEn differentiation. Therefore, we examined the stage-specific role of SOX17 in ExEn differentiation. Ad-SOX17 transduction was performed in human ESCs treated with BMP4 for 0, 1, 2, 3, or 4 days, and the Ad-SOX17-transduced cells were cultured with medium containing BMP4 until day 5 (Figures 1A–1D). We confirmed the expression of exogenous SOX17 in the human ESC-derived mesendoderm cells transduced with Ad-SOX17 (Figure S3). Since BMP4 is known for its capability to induce both ExEn and trophectoderm [8], [9], we analyzed not only the expression levels of ExEn markers but also those of trophectoderm markers by real-time RT-PCR after 5 days of differentiation (Figures 1A and 1B). The transduction of Ad-SOX17 on day 1 led to the highest expression levels of ExEn markers, alpha-fetoprotein (AFP), GATA4, laminin B1 (LAMB1), and SOX7 [17], [18], [19]. In contrast, the expression levels of the trophectoderm markers CDX2, GATA2, hCGα (human chorionic gonadotropin), and hCGβ [20] were down-regulated in Ad-SOX17-transduced cells as compared with non-transduced cells (Figure 1B). The expression levels of the pluripotent marker NANOG and DE marker GSC were not increased by SOX17 transduction (Figures 1C and 1D). We confirmed that there were no differences between non-transduced cells and Ad-LacZ-transduced cells in gene expression levels of all the markers investigated in Figures 1A–1D (data not shown). Therefore, we concluded that ExEn cells were efficiently induced from Ad-SOX17-transduced PrE cells.

The effects of SOX17 transduction on the ExEn differentiation from human ESC-derived PrE cells were also assessed by quantifying AFP- or SOX7-positive ExEn cells. The percentage of AFP- or SOX7-positive cells was significantly increased in Ad-SOX17-transduced cells (69.7% and 63.3%, respectively) (Figure 1E). Similar results were observed in the human iPS cell lines (201B7, Dotcom, and Tic) (Figure 1F). These findings indicated that stage-specific SOX17 overexpression in human ESC-derived PrE cells enhances ExEn differentiation.

### Mesendoderm stage-specific SOX17 overexpression promotes directive DE differentiation from human ESCs

**Figure 2:**
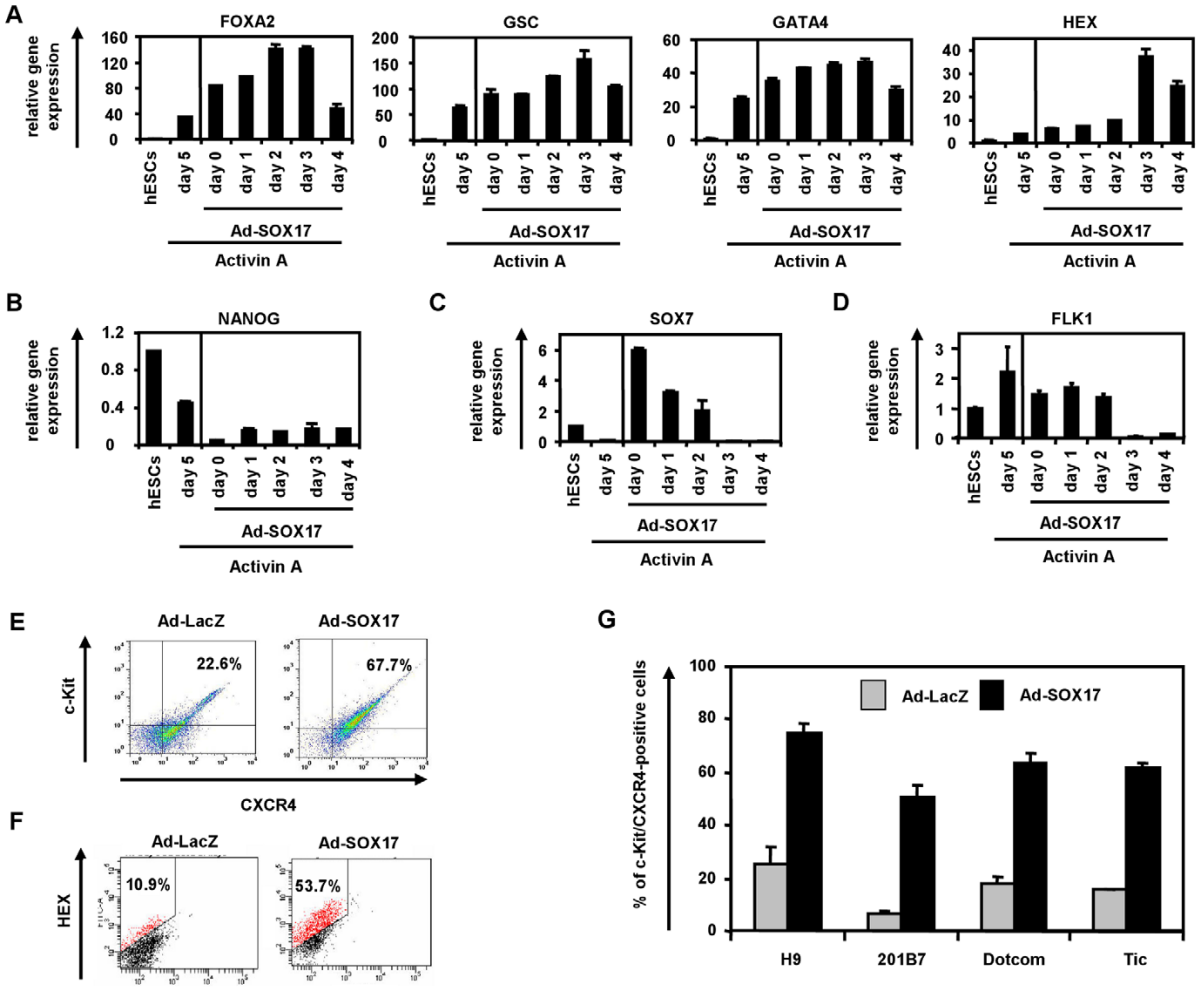
Efficient DE differentiation from human ESC- and iPSC-derived mesendoderm cells by SOX17 transduction. (A–D) Undifferentiated human ESCs (H9) and Activin A-induced human ESC-derived cells, which were cultured with the medium containing Activin A (100 ng/ml) for 0, 1, 2, 3, and 4 days, were transduced with 3,000 VP/cell of Ad-SOX17 for 1.5 h. Ad-SOX17-transduced cells were cultured with 100 ng/ml of Activin A, and the gene expression levels of (A) the DE markers (FOXA2, GSC, and GATA4) and anterior DE marker (HEX), (B) the pluripotent marker (NANOG), (C) the ExEn marker (SOX7), and (D) the mesoderm marker (FLK1) were examined by real-time RT-PCR on day 5 of differentiation. The horizontal axis represents the day on which the cells were transduced with Ad-SOX17. The expression levels of human ESCs on day 0 were defined 1.0. (E, F) After human ESCs were cultured with 100 ng/ml of Activin A for 3 days, human ESC-derived mesendoderm cells were transduced with Ad-LacZ or Ad-SOX17 and cultured until day 5. Ad-LacZ- or Ad-SOX17-transduced DE cells were subjected to immunostaining with anti-c-Kit, anti-CXCR4 (E) and anti-HEX antibodies (F) and then analyzed by flow cytometry. (G) After Ad-LacZ or Ad-SOX17 transduction, the DE differentiation efficacies of the human ES cell line (H9) and three human iPS cell lines (201B7, Dotcom, and Tic) were compared at day 5 of differentiation. All data are represented as the means±SD (*n = *3).

To examine the effects of transient SOX17 overexpression on DE differentiation from human ESCs, we optimized the timing of the Ad-SOX17 transduction. Ad-SOX17 transduction was performed in human ESCs treated with Activin A (100 ng/ml) for 0, 1, 2, 3, or 4 days, and the Ad-SOX17-transduced cells were cultured with medium containing Activin A (100 ng/ml) until day 5 (Figures 2A–2C). Using a fiber-modified Ad vector, both undifferentiated human ESCs and Activin A-induced human ESC-derived cells were efficiently transduced (Figure S4). The transduction of SOX17 on day 3 led to the highest expression levels of the DE markers FOXA2 [21], GSC [22], GATA4 [17], and HEX [23] (Figure 2A). In contrast to the DE markers, the expression levels of the pluripotent marker NANOG [24] were down-regulated in Ad-SOX17-transduced cells as compared with non-transduced cells (Figure 2B). The expression levels of the ExEn marker SOX7 [14] were up-regulated, when Ad-SOX17 transduction was performed into human ESCs treated with Activin A (100 ng/ml) for 0, 1, or 2 days (Figure 2C). On the other hand, the expression levels of the ExEn marker SOX7 were significantly down-regulated, when Ad-SOX17 transduction was performed into human ESCs treated with Activin A (100 ng/ml) for 3 or 4 days, indicating that SOX17 overexpression prior to mesendoderm formation (day 0, 1, and 2) promoted not only DE differentiation but also ExEn differentiation. Similar results were obtained with the human iPS cell line (Tic) (Figure S5). Although the expression levels of the mesoderm marker FLK1 [25] did not exhibit any change when Ad-SOX17 transduction was performed into human ESCs treated with Activin A (100 ng/ml) for 0, 1, or 2 days (Figure 2D), their expression levels were significantly down-regulated when Ad-SOX17 transduction was performed into human ESCs treated with Activin A (100 ng/ml) for 3 or 4 days. These results suggest that SOX17 overexpression promotes directive differentiation from mesendoderm cells into the DE cells, but not into mesoderm cells. We also confirmed that Ad-vector mediated gene expression in the human ESC-derived mesendoderm cells (day 3) continued until day 6 and disappeared on day 10 (Figure S6). SOX17 transduction in the human ESC-derived cells on day 3 and 4 had no effect on cell viability, while that in the cells on day 0, 1, and 2 resulted in severely impaired cell viability (Figure S7), probably because SOX17 transduction directed the cells on day 0, 1, and 2 to differentiate into ExEn cells but the medium containing Activin A (100 ng/ml) was inappropriate for ExEn cells. We confirmed that there were no differences between non-transduced cells and Ad-LacZ-transduced cells in gene expression levels of all the markers investigated in Figures 2A–2D (data not shown). These results indicated that stage-specific SOX17 overexpression in human ESC-derived mesendoderm cells is essential for promoting efficient DE differentiation.

It has been previously reported that human ESC-derived mesendoderm cells and DE cells became CXCR4-positive (>80%) by culturing human ESCs with Activin A (100 ng/ml) [26]. However, Activin A is not sufficient for homogenous differentiation of c-Kit/CXCR4-double-positive DE cells [10], [11] or HEX-positive anterior DE cells [23]. Seguin et al. and Morrison et al. reported that the differentiation efficiency of c-Kit/CXCR4-double-positive DE cells was approximately 30% in the absence of stable Sox17 expression and that of HEX-positive anterior DE cells was only about 10% [10], [23]. Therefore, we next examined whether Ad-SOX17 transduction improves the differentiation efficiency of c-Kit/CXCR4-double-positive DE cells and HEX-positive anterior DE cells. Human ESC-derived mesendoderm cells were transduced with Ad-SOX17, and the number of CXCR4/c-Kit-double-positive cells was analyzed by using a flow cytometer. The percentage of CXCR4/c-Kit-double-positive cells was significantly increased in Ad-SOX17-transduced cells (67.7%), while that in Ad-LacZ-transduced cells was only 22% (Figure 2E). The percentage of HEX-positive cells was also significantly increased in Ad-SOX17-transduced cells (53.7%), while that in Ad-LacZ-transduced cells was approximately 11% (Figure 2F). Similar results were also observed in the three human iPS cell lines (201B7, Dotcom, and Tic) (Figure 2G). These findings indicated that stage-specific SOX17 overexpression in human ESC-derived mesendoderm cells promotes efficient differentiation of DE cells.

### Ad-SOX17-transduced cells tend to differentiate into the hepatic lineage

**Figure 3:**
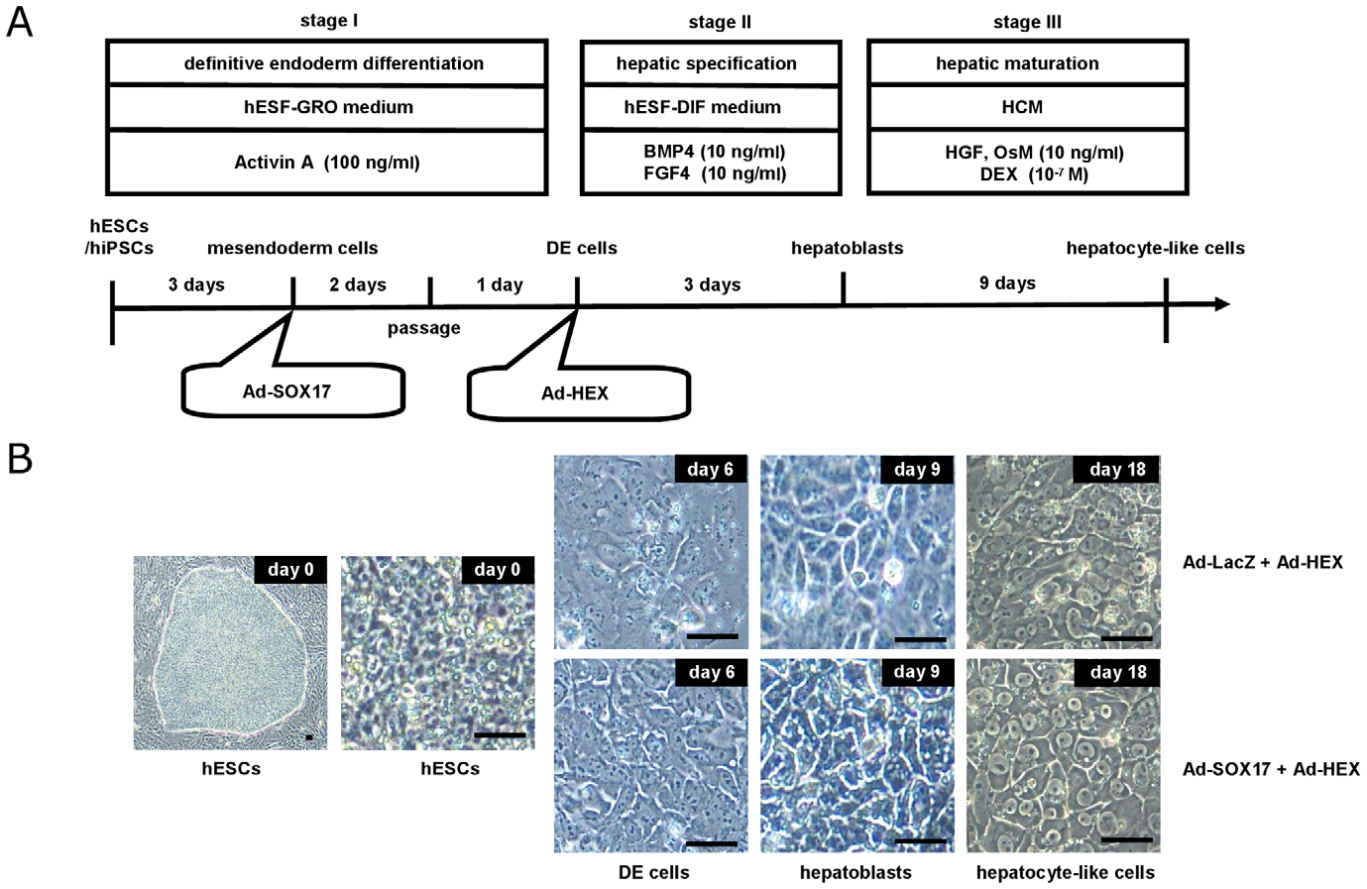
Hepatic Differentiation of Human ESC- and iPSC-Derived DE Cells Transduced with Ad-HEX. (A) The procedure for differentiation of human ESCs and iPSCs into hepatoblasts and hepatocyte-like cells is presented schematically. Both hESF-GRO and hESF-DIF medium were supplemented with 5 factors and 0.5 mg/ml fatty acid-free BSA, as described in the Materials and Methods section. (B) Sequential morphological changes (day 0–18) of human ESCs (H9) differentiated into hepatocyte-like cells via the DE cells and the hepatoblasts are shown. The scale bar represents 50 µm.

**Figure 4:**
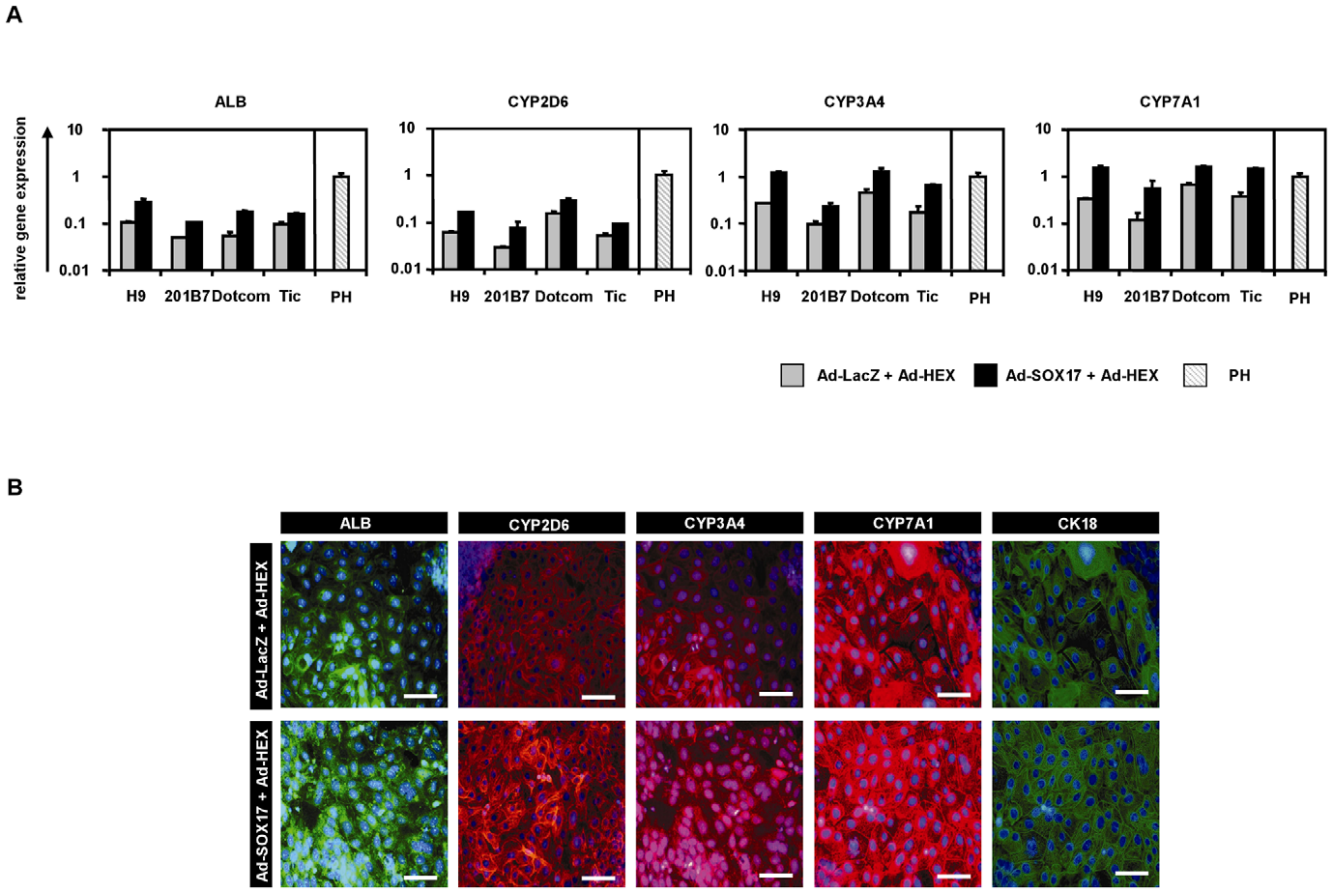
Characterization of hepatocyte-like cells from human ESC- and iPSC-derived DE cells. (A) The Ad-LacZ-transduced cells and Ad-SOX17-transduced cells were transduced with 3,000 VP/cell of Ad-HEX for 1.5 h on day 6. On day 18 of differentiation, the levels of expression of the hepatocyte markers (ALB, CYP2D6, CYP3A4, and CYP7A1) were examined by real-time RT-PCR in human ESC (H9)-derived hepatocyte-like cells and human iPSC (201B7, Dotcom, or Tic)-derived hepatocyte-like cells. The gene expression profiles of cells transduced with both Ad-SOX17 and Ad-HEX (black bar) were compared with those of cells transduced with both Ad-LacZ and Ad-HEX (gray bar). The expression level of primary human hepatocytes (PH, hatched bar), which were cultured 48 h after plating the cells, were defined as 1.0. All data are represented as the means±SD (*n = *3). (B) The expression of the hepatocyte markers ALB (green), CYP2D6 (red), CYP3A4 (red), CYP7A1 (red), and CK18 (green) was also examined by immunohistochemistry on day 18 of differentiation. Nuclei were counterstained with DAPI (blue). The scale bar represents 50 µm.

To investigate whether Ad-SOX17-transduced cells have the ability to differentiate into hepatoblasts and hepatocyte-like cells, Ad-SOX17-transduced cells were differentiated according to our previously described method [13]. Our previous report demonstrated that transient HEX transduction efficiently generates hepatoblasts from human ESC- and iPSC-derived DE cells. The hepatic differentiation protocol used in this study is illustrated in Figure 3A. After the hepatic differentiation, the morphology of human ESCs transduced with Ad-SOX17 followed by Ad-HEX was gradually changed into a hepatocyte morphology: polygonal in shape with distinct round nuclei by day 18 (Figure 3B). We also examined hepatic gene and protein expression levels on day18 of differentiation. For this purpose, we used a human ES cell line (H9) and three human iPS cell lines (201B7, Dotcom, Tic). On day 18 of differentiation, the gene and protein expression analysis showed up-regulation of the hepatic markers albumin (ALB) [27], cytochrome P450 2D6 (CYP2D6), CYP3A4, and CYP7A1 [28] mRNA and ALB, CYP2D6, CYP3A4, CYP7A1, and cytokeratin (CK)18 proteins in both Ad-SOX17- and Ad-HEX-transduced cells transduced cells as compared with both Ad-LacZ- and Ad-HEX-transduced cells (Figures 4A and 4B). These results indicated that Ad-SOX17-transduced cells were more committed to the hepatic lineage than non-transduced cells.

## Discussion

The directed differentiation from human ESCs and iPSCs is a useful model system for studying mammalian development as well as a powerful tool for regenerative medicine [29]. In the present study, we elucidated the bidirectional role of SOX17 on either ExEn or DE differentiation from human ESCs and iPSCs. We initially confirmed that initiation of SOX17 expression was consistent with the time period of PrE or mesendoderm cells formation (Figures S1 and S2). We speculated that stage-specific transient SOX17 transduction in PrE or mesendoderm could enhance ExEn or DE differentiation from human ESCs and iPSCs, respectively.

SOX17 transduction at the pluripotent stage promoted random differentiation giving heterogeneous populations containing both ExEn and DE cells were obtained (Figures 2A–2C). Qu et al. reported that SOX17 promotes random differentiation of mouse ESCs into PrE cells and DE cells *in vitro* [30], which is in consistent with the present study. Previously, Niakan et al. and Seguin et al. respectively demonstrated that ESCs could promote either ExEn or DE differentiation by stable SOX17 expression, respectively [10], [12]. Although these discrepancies might be attributable to differences in the species used in the experiments (i.e., human versus mice), SOX17 might have distinct functions according to the appropriate differentiation stage. To elucidate these discrepancies, we examined the stage-specific roles of SOX17 in the present study, and found that human ESCs and iPSCs could differentiate into either ExEn or DE cells when SOX17 was overexpressed at the PrE or mesendoderm stage, respectively, but not when it was overexpressed at the pluripotent stage (Figures 1 and 2). This is because endogenous SOX17 is strongly expressed in the PrE and primitive streak tissues but only slightly expressed in the inner cell mass, our system might adequately reflect the early embryogenesis [14], [31].

In ExEn differentiation from human ESCs, stage-specific SOX17 overexpression in human ESC-derived PrE cells promoted efficient ExEn differentiation and repressed trophectoderm differentiation (Figures 1A and 1B), although SOX17 transduction at the pluripotent stage did not induce the efficient differentiation of ExEn cells. In our protocol, the stage-specific overexpression of SOX17 could elevate the efficacy of AFP-positive or SOX7-positive ExEn differentiation from human ESCs and iPSCs. The reason for the efficient ExEn differentiation by SOX17 transduction might be due to the fact that SOX17 lies downstream from GATA6 and directly regulates the expression of GATA4 and GATA6 [12]. Although it was previously been reported that Sox17 plays a substantial role in late-stage differentiation of ExEn cells *in vitro* [32], those reports utilized embryoid body formation, in which other types of cells, including endoderm, mesoderm, and ectoderm cells, might have influences on cellular differentiation. The present study showed the role of SOX17 in a homogeneous differentiation system by utilizing a mono-layer culture system.

In DE differentiation from human ESCs, we found that DE cells were efficiently differentiated from the human ESC-derived mesendoderm cells by stage-specific SOX17 overexpression (Figure 2). Therefore, we concluded that SOX17 plays a significant role in the differentiation of mesendoderm cells to DE cells. Although SOX17 overexpression before the formation of mesendoderm cells did not affect mesoderm differentiation, SOX17 transduction at the mesendoderm stage selectively promoted DE differentiation and repressed mesoderm differentiation (Figures 2A and 2D). These results show that SOX17 plays a crucial role in decision of DE differentiation from mesendoderm cells, as previous studies suggested [33], [34]. Interestingly, SOX17 transduction at the pluripotent stage promoted not only DE differentiation but also ExEn differentiation even in the presence of Activin A (Figures 2A and 2C), demonstrating that transduction at an inappropriate stage of differentiation prevents directed differentiation. These results suggest that stage-specific SOX17 transduction mimicking the gene expression pattern in embryogenesis could selectively promote DE differentiation.

Another important finding about DE differentiation is that the protocol in the present study was sufficient for nearly homogeneous DE and anterior DE differentiation by mesendoderm stage-specific SOX17 overexpression; the differentiation efficacies of c-Kit/CXCR4-double-positive DE cells and HEX-positive anterior DE cells were approximately 70% and 54%, respectively (Figures 2E and 2F). The conventional differentiation protocols without gene transfer were not sufficient for homogenous DE and anterior DE differentiation; the differentiation efficacies of DE and anterior DE were approximately 30% and 10%, respectively [10], [11], [23]. One of the reasons for the efficient DE differentiation by SOX17 transduction might be the activation of the FOXA2 gene which could regulate many endoderm-associated genes [35]. Moreover, SOX17-transduced cells were more committed to the hepatic lineage (Figure 4). This might be because the number of HEX-positive anterior DE cell populations was increased by SOX17 transduction. Recent studies have shown that the conditional expression of Sox17 in the pancreas at E12.5, when it is not normally expressed, is sufficient to promote biliary differentiation at the expense of endocrine cells [36]. Therefore, we reconfirmed that our protocol in which SOX17 was transiently transduced at the appropriate stage of differentiation was useful for DE and hepatic differentiation from human ESCs and iPSCs.

Using human iPSCs as well as human ESCs, we confirmed that stage-specific overexpression of SOX17 could promote directive differentiation of either ExEn or DE cells (Figures 1F, 2G, and 4A). Interestingly, a difference of DE and hepatic differentiation efficacy among human iPS cell lines was observed (Figures 1F and 2G). Therefore, it would be necessary to select a human iPS cell line that is suitable for hepatic differentiation in the case of medical applications, such as liver transplantation.

To control cellular differentiation mimicking embryogenesis, we employed Ad vectors, which are one of the most efficient transient gene delivery vehicles and have been widely used in both experimental studies and clinical trials [37]. Recently, we have also demonstrated that ectopic HEX expression by Ad vectors in human ESC-derived DE cells markedly enhances the hepatic differentiation [13]. Thus, Ad vector-mediated transient gene transfer should be a powerful tool for regulating cellular differentiation.

In summary, the findings presented here demonstrate a stage-specific role of SOX17 in the ExEn and DE differentiation from human ESCs and iPSCs (Figure S8). Although previous reports showed that SOX17 overexpression in ESCs leads to differentiation of either ExEn or DE cells, we established a novel method to promote directive differentiation by SOX17 transduction. Because we utilized a stage-specific overexpression system, our findings provide further evidence that the lineage commitment in this method seems to reflects what is observed in embryonic development. In the present study, both human ESCs and iPSCs (3 lines) were used and all cell lines showed efficient ExEn or DE differentiation, indicating that our novel protocol is a powerful tool for efficient and cell line-independent endoderm differentiation. Moreover, the establishing methods for efficient hepatic differentiation by sequential SOX17 and HEX transduction would be useful for *in vitro* applications such as screening of pharmacological compounds as well as for regenerative therapy.

## Materials and Methods

### *In vitro* Differentiation

Before the initiation of cellular differentiation, the medium of human ESCs and iPSCs was exchanged for a defined serum-free medium hESF9 [38] and cultured as we previously reported. hESF9 consists of hESF-GRO medium (Cell Science & Technology Institute) supplemented with 5 factors (10 µg/ml human recombinant insulin, 5 µg/ml human apotransferrin, 10 µM 2-mercaptoethanol, 10 µM ethanolamine, and 10 µM sodium selenite), oleic acid conjugated with fatty acid free bovine albumin, 10 ng/ml FGF2, and 100 ng/ml heparin (all from Sigma).

To induce, ExEn cells, human ESCs and iPSCs were cultured for 5 days on a gelatin-coated plate in mouse embryonic conditioned-medium supplemented with 20 ng/ml BMP4 (R&D system) and 1% FCS (GIBCO-BRL).

The differentiation protocol for induction of DE cells, hepatoblasts, and hepatocyte-like cells was based on our previous report with some modifications [13]. Briefly, in DE differentiation, human ESCs and iPSCs were cultured for 5 days on a Matrigel (BD)-coated plate in hESF-DIF medium (Cell Science & Technology Institute) supplemented with the above-described 5 factors, 0.5 mg/ml BSA, and 100 ng/ml Activin A (R&D Systems). For induction of hepatoblasts, the DE cells were transduced with 3,000 VP/cell of Ad-HEX for 1.5 h and cultured in hESF-DIF (Cell Science & Technology Institute) medium supplemented with the above-described 5 factors, 0.5 mg/ml BSA, 10 ng/ml bone morphology protein 4 (BMP4) (R&D Systems), and 10 ng/ml FGF4 (R&D systems). In hepatic differentiation, the cells were cultured in hepatocyte culture medium (HCM) supplemented with SingleQuots (Lonza), 10 ng/ml hepatocyte growth factor (HGF) (R&D Systems), 10 ng/ml Oncostatin M (OsM) (R&D Systems), and 10^−7^ M dexamethasone (DEX) (Sigma).

### Human ESC and iPSC Culture

A human ES cell line, H9 (WiCell Research Institute), was maintained on a feeder layer of mitomycin C-treated mouse embryonic fibroblasts (Millipore) with Repro Stem (Repro CELL), supplemented with 5 ng/ml fibroblast growth factor 2 (FGF2) (Sigma). Human ESCs were dissociated with 0.1 mg/ml dispase (Roche Diagnostics) into small clumps, and subcultured every 4 or 5 days. Two human iPS cell lines generated from the human embryonic lung fibroblast cell line MCR5 were provided from the JCRB Cell Bank (Tic, JCRB Number: JCRB1331; and Dotcom, JCRB Number: JCRB1327) [39], [40]. These human iPS cell lines were maintained on a feeder layer of mitomycin C-treated mouse embryonic fibroblasts with iPSellon (Cardio), supplemented with 10 ng /ml FGF2. Another human iPS cell line, 201B7, generated from human dermal fibroblasts (HDF) was kindly provided by Dr. S. Yamanaka (Kyoto University) [6]. The human iPS cell line 201B7 was maintained on a feeder layer of mitomycin C-treated mouse embryonic fibroblasts with Repro Stem (Repro CELL), supplemented with 5 ng/ml FGF2 (Sigma). Human iPSCs were dissociated with 0.1 mg/ml dispase (Roche Diagnostics) into small clumps, and subcultured every 5 or 6 days.

### Adenovirus (Ad) Vectors

Ad vectors were constructed by an improved *in vitro* ligation method [41], [42]. The human SOX17 gene (accession number NM_022454) was amplified by PCR using primers designed to incorporate the 5′ BamHI and 3′ XbaI restriction enzyme sites: Fwd 5′-gcagggatccagcgccatgagcagcccgg-3′ and Rev 5′-cttctagagatcagggacctgtcacacgtc-3′. The human SOX17 gene was inserted into pcDNA3 (Invitrogen), resulting in pcDNA-SOX17, and then the human SOX17 gene was inserted into pHMEF5 [15], which contains the human EF-1α promoter, resulting in pHMEF-SOX17. The pHMEF-SOX17 was digested with I-CeuI/PI-SceI and ligated into I-CeuI/PI-SceI-digested pAdHM41-K7 [16], resulting in pAd-SOX17. The human elongation factor-1α (EF-1α) promoter-driven LacZ- or HEX-expressing Ad vectors, Ad-LacZ or Ad-HEX, were constructed previously. [13], [43]. Ad-SOX17, Ad-HEX, and Ad-LacZ, which contain a stretch of lysine residue (K7) peptides in the C-terminal region of the fiber knob for more efficient transduction of human ESCs, iPSCs, and DE cells, were generated and purified as described previously [13], [15], [43]. The vector particle (VP) titer was determined by using a spectrophotometric method [44].

### Flow Cytometry

Single-cell suspensions of human ESCs, iPSCs, and their derivatives were fixed with methanol at 4°C for 20 min, then incubated with the primary antibody, followed by the secondary antibody. Flow cytometry analysis was performed using a FACS LSR Fortessa flow cytometer (Becton Dickinson).

### RNA Isolation and Reverse Transcription-Polymerase Chain Reaction (RT-PCR)

Total RNA was isolated from human ESCs, iPSCs, and their derivatives using ISOGENE (Nippon Gene) according to the manufacture's instructions. Primary human hepatocytes were purchased from CellzDirect. cDNA was synthesized using 500 ng of total RNA with a Superscript VILO cDNA synthesis kit (Invitrogen). Real-time RT-PCR was performed with Taqman gene expression assays (Applied Biosystems) or SYBR Premix Ex Taq (TaKaRa) using an ABI PRISM 7000 Sequence Detector (Applied Biosystems). Relative quantification was performed against a standard curve and the values were normalized against the input determined for the housekeeping gene, glyceraldehyde 3-phosphate dehydrogenase (GAPDH). The primer sequences used in this study are described in Table S1.

### Immunohistochemistry

The cells were fixed with methanol or 4% PFA. After blocking with PBS containing 2% BSA and 0.2% Triton X-100 (Sigma), the cells were incubated with primary antibody at 4°C for 16 h, followed by incubation with a secondary antibody that was labeled with Alexa Fluor 488 or Alexa Fluor 594 (Invitrogen) at room temperature for 1 h. All the antibodies are listed in Table S2.

### Crystal Violet Staining

The human ESC-derived cells that had adhered to the wells were stained with 200 µl of 0.3% crystal violet solution at room temperature for 15 min. Excess crystal violet was then removed and the wells were washed three times. Fixed crystal violet was solubilized in 200 µl of 100% ethanol at room temperature for 15 min. Cell viability was estimated by measuring the absorbance at 595 nm of each well using a microtiter plate reader (Sunrise, Tecan).

### LacZ Assay

The human ESC- and iPSC-derived cells were transduced with Ad-LacZ at 3,000 VP/cell for 1.5 h. After culturing for the indicated number of days, 5-bromo-4-chloro-3-indolyl β-D-galactopyranoside (X-Gal) staining was performed as described previously [15].

## Supporting Information

Table S1 **List of Taqman probes and primers used in this study.**
(DOC)

Table S2 **List of antibodies used in this study.**
(DOC)

Figure S1 **PrE cells formation from human ESCs on day 1 of differentiation.** (A) The procedure for differentiation of human ESCs and iPSCs to ExEn cells by treatment with BMP4 (20 ng/ml) is presented schematically. (B) Human ESCs (H9) were morphologically changed during ExEn differentiation; when human ESCs were cultured with the medium containing BMP4 (20 ng/ml) for 5 days, the cells began to show flattened epithelial morphology. The scale bar represents 50 µm. (C–E) The tTemporal protein expression analysis during ExEn differentiation was performed by immunohistochemistry. The PrE markers COUP-TF1 [21] (red), SOX17 [14] (red), and SOX7 [14] (red) were detected on day 1. In contrast to the PS markers, the expression of the DE marker GSC [22] (red) was not detected and the level of the pluripotent marker NANOG (green) declined between day 0 and day 1. Nuclei were counterstained with DAPI (blue). The scale bar represents 50 µm.
(PDF)

Figure S2 **Mesendoderm cells formation from human ESCs on day 3 of differentiation.** (A) The procedure for differentiation of human ESCs and iPSCs to DE cells by treatment with Activin A (100 ng/ml) is presented schematically. hESF-GRO medium was supplemented with 5 factors and 0.5 mg/ml fatty acid free BSA, as described in the Materials and Methods. (B) Human ESCs (H9) were morphologically changed during DE differentiation; when human ESCs were cultured with the medium containing Activin A (100 ng/ml) for 5 days, the morphology of the cells began to show visible cell-cell boundaries. The scale bar represents 50 µm. (C–E) The tTemporal protein expression analysis during DE differentiation was performed by immunohistochemistry. The anterior PS markers FOXA2 [21] (red), GSC [22] (red), and SOX17 [14] (red) were adequately detected on day 3. The PS marker T [45] (red) was detected until day 3. In contrast to the PS markers, the expression of the pluripotent marker NANOG [24] (green) declined between day 2 and day 3. Nuclei were counterstained with DAPI (blue). The scale bar represents 50 µm.
(PDF)

Figure S3 **Overexpression of SOX17 mRNA in human ESC (H9)-derived PS cells by Ad-SOX17 transduction.** Human ESC-derived PS cells (day 1) were transduced with 3,000VP/cell of Ad-SOX17 for 1.5 h. On day 3 of differentiation, real-time RT-PCR analysis of the SOX17 expression was performed in Ad-LacZ-transduced cells and Ad-SOX17-transduced cells. On the y axis, the expression levels of undifferentiated human ESCs on day 0 was were taken defined as 1.0. All data are represented as the means±SD (*n = *3).
(PDF)

Figure S4 **Efficient transduction in Activin A-induced human ESC (H9)-derived cells by using a fiber-modified Ad vector containing the EF-1α promoter.** Undifferentiated human ESCs and Activin A-induced human ESC-derived cells, which were cultured with the medium containing Activin A (100 ng/ml) for 0, 1, 2, 3, and 4 days, were transduced with 3,000 vector particles (VP)/cell of Ad-LacZ for 1.5 h. The day after transduction, X-gal staining was performed. The scale bar represents 100 µm. Similar results were obtained in two independent experiments.
(PDF)

Figure S5 **Optimization of the time period for Ad-SOX17 transduction to promote DE differentiation from human iPSCs (Tic).** Undifferentiated human iPSCs and Activin A-induced human iPSC-derived cells, which were cultured with the medium containing Activin A (100 ng/ml) for 0, 1, 2, 3, and 4 days, were transduced with 3,000 VP/cell of Ad-SOX17 for 1.5 h. Ad-SOX17-transduced cells were cultured with Activin A (100 ng/ml) until day 5, and then real-time RT-PCR analysis was performed. The horizontal axis represents the day on which the cells were transduced with Ad-SOX17. On the y axis, the expression levels of undifferentiated cells on day 0 was were taken defined as 1.0. All data are represented as the means±SD (*n = *3).
(PDF)

Figure S6 **Time course of LacZ expression in human ESC (H9)-derived mesendoderm cells transduced with Ad-LacZ.** The hHuman ESC-derived mesendoderm cells (day 3) were transduced with 3,000 VP/cell of Ad-LacZ for 1.5 h. On days 4, 5, 6, 8, and 10, X-gal staining was performed. Note that human ESC-derived cells were passaged on day 5. The scale bar represents 100 µm. Similar results were obtained in two independent experiments.
(PDF)

Figure S7 **Optimization of the time period for Ad-SOX17 transduction into Activin A-induced human ESC (H9)-derived cells.** Undifferentiated human ESCs and Activin A-induced hESC-derived cells, which were cultured with the medium containing Activin A (100 ng/ml) for 0, 1, 2, 3, and 4 days, were transduced with 3,000 VP/cell of Ad-LacZ or Ad-SOX17 for 1.5 h. Ad-SOX17-transduced cells were cultured with Activin A (100 ng/ml) until day 5, then the cell viability was evaluated with crystal violet staining. The horizontal axis represents the day on which the cells were transduced with Ad-SOX17. On the y axis, the level of non-transduced cells was taken defined as 1.0. All data are represented as the means±SD (n = 3).
(PDF)

Figure S8 **Model of differentiation of human ESCs and iPSCs into ExEn and DE cells by stage-specific SOX17 transduction.** The ExEn and DE differentiation process is divided into at least two stages. In the first stage, human ESCs differentiate into either PrE cells by treatment with BMP4 (20 ng/ml) or mesendoderm cells by treatment with Activin A (100 ng/ml). In the second stage, SOX17 promotes the further differentiation of each precursor cell into ExEn and DE cells, respectively. We have demonstrated that the efficient differentiation of these two distinct endoderm lineages is accomplished by stage-specific SOX17 transduction.
(PDF)

## References

[1] Enders AC, Given RL, Schlafke S (1978). Differentiation and migration of endoderm in the rat and mouse at implantation.. Anat Rec.

[2] Gardner RL (1983). Origin and differentiation of extraembryonic tissues in the mouse.. Int Rev Exp Pathol.

[3] Grapin-Botton A, Constam D (2007). Evolution of the mechanisms and molecular control of endoderm formation.. Mech Dev.

[4] Tam PP, Kanai-Azuma M, Kanai Y (2003). Early endoderm development in vertebrates: lineage differentiation and morphogenetic function.. Curr Opin Genet Dev.

[5] Thomson JA, Itskovitz-Eldor J, Shapiro SS, Waknitz MA, Swiergiel JJ (1998). Embryonic stem cell lines derived from human blastocysts.. Science.

[6] Takahashi K, Tanabe K, Ohnuki M, Narita M, Ichisaka T (2007). Induction of pluripotent stem cells from adult human fibroblasts by defined factors.. Cell.

[7] Yu J, Vodyanik MA, Smuga-Otto K, Antosiewicz-Bourget J, Frane JL (2007). Induced pluripotent stem cell lines derived from human somatic cells.. Science.

[8] Xu RH, Chen X, Li DS, Li R, Addicks GC (2002). BMP4 initiates human embryonic stem cell differentiation to trophoblast.. Nat Biotechnol.

[9] Pera MF, Andrade J, Houssami S, Reubinoff B, Trounson A (2004). Regulation of human embryonic stem cell differentiation by BMP-2 and its antagonist noggin.. J Cell Sci.

[10] Seguin CA, Draper JS, Nagy A, Rossant J (2008). Establishment of endoderm progenitors by SOX transcription factor expression in human embryonic stem cells.. Cell Stem Cell.

[11] Gouon-Evans V, Boussemart L, Gadue P, Nierhoff D, Koehler CI (2006). BMP-4 is required for hepatic specification of mouse embryonic stem cell-derived definitive endoderm.. Nat Biotechnol.

[12] Niakan KK, Ji H, Maehr R, Vokes SA, Rodolfa KT (2010). Sox17 promotes differentiation in mouse embryonic stem cells by directly regulating extraembryonic gene expression and indirectly antagonizing self-renewal.. Genes Dev.

[13] Inamura M, Kawabata K, Takayama K, Tashiro K, Sakurai F (2011). Efficient Generation of Hepatoblasts From Human ES Cells and iPS Cells by Transient Overexpression of Homeobox Gene HEX.. Mol Ther.

[14] Kanai-Azuma M, Kanai Y, Gad JM, Tajima Y, Taya C (2002). Depletion of definitive gut endoderm in Sox17-null mutant mice.. Development.

[15] Kawabata K, Sakurai F, Yamaguchi T, Hayakawa T, Mizuguchi H (2005). Efficient gene transfer into mouse embryonic stem cells with adenovirus vectors.. Mol Ther.

[16] Koizumi N, Mizuguchi H, Utoguchi N, Watanabe Y, Hayakawa T (2003). Generation of fiber-modified adenovirus vectors containing heterologous peptides in both the HI loop and C terminus of the fiber knob.. J Gene Med.

[17] Fujikura J, Yamato E, Yonemura S, Hosoda K, Masui S (2002). Differentiation of embryonic stem cells is induced by GATA factors.. Genes Dev.

[18] Koutsourakis M, Langeveld A, Patient R, Beddington R, Grosveld F (1999). The transcription factor GATA6 is essential for early extraembryonic development.. Development.

[19] Morrisey EE, Tang Z, Sigrist K, Lu MM, Jiang F (1998). GATA6 regulates HNF4 and is required for differentiation of visceral endoderm in the mouse embryo.. Genes Dev.

[20] Kunath T, Strumpf D, Rossant J (2004). Early trophoblast determination and stem cell maintenance in the mouse-–a review.. Placenta.

[21] Sasaki H, Hogan BL (1993). Differential expression of multiple fork head related genes during gastrulation and axial pattern formation in the mouse embryo.. Development.

[22] Blum M, Gaunt SJ, Cho KW, Steinbeisser H, Blumberg B (1992). Gastrulation in the mouse: the role of the homeobox gene goosecoid.. Cell.

[23] Morrison GM, Oikonomopoulou I, Migueles RP, Soneji S, Livigni A (2008). Anterior definitive endoderm from ESCs reveals a role for FGF signaling.. Cell Stem Cell.

[24] Mitsui K, Tokuzawa Y, Itoh H, Segawa K, Murakami M (2003). The homeoprotein Nanog is required for maintenance of pluripotency in mouse epiblast and ES cells.. Cell.

[25] Shalaby F, Rossant J, Yamaguchi TP, Gertsenstein M, Wu XF (1995). Failure of blood-island formation and vasculogenesis in Flk-1-deficient mice.. Nature.

[26] D'Amour KA, Agulnick AD, Eliazer S, Kelly OG, Kroon E (2005). Efficient differentiation of human embryonic stem cells to definitive endoderm.. Nat Biotechnol.

[27] Shiojiri N (1984). The origin of intrahepatic bile duct cells in the mouse.. J Embryol Exp Morphol.

[28] Ingelman-Sundberg M, Oscarson M, McLellan RA (1999). Polymorphic human cytochrome P450 enzymes: an opportunity for individualized drug treatment.. Trends Pharmacol Sci.

[29] Murry CE, Keller G (2008). Differentiation of embryonic stem cells to clinically relevant populations: lessons from embryonic development.. Cell.

[30] Qu XB, Pan J, Zhang C, Huang SY (2008). Sox17 facilitates the differentiation of mouse embryonic stem cells into primitive and definitive endoderm in vitro.. Dev Growth Differ.

[31] Sherwood RI, Jitianu C, Cleaver O, Shaywitz DA, Lamenzo JO (2007). Prospective isolation and global gene expression analysis of definitive and visceral endoderm.. Dev Biol.

[32] Shimoda M, Kanai-Azuma M, Hara K, Miyazaki S, Kanai Y (2007). Sox17 plays a substantial role in late-stage differentiation of the extraembryonic endoderm in vitro.. J Cell Sci.

[33] Yasunaga M, Tada S, Torikai-Nishikawa S, Nakano Y, Okada M (2005). Induction and monitoring of definitive and visceral endoderm differentiation of mouse ES cells.. Nat Biotechnol.

[34] Gadue P, Huber TL, Paddison PJ, Keller GM (2006). Wnt and TGF-beta signaling are required for the induction of an in vitro model of primitive streak formation using embryonic stem cells.. Proc Natl Acad Sci U S A.

[35] Levinson-Dushnik M, Benvenisty N (1997). Involvement of hepatocyte nuclear factor 3 in endoderm differentiation of embryonic stem cells.. Mol Cell Biol.

[36] Spence JR, Lange AW, Lin SC, Kaestner KH, Lowy AM (2009). Sox17 regulates organ lineage segregation of ventral foregut progenitor cells.. Dev Cell.

[37] Mizuguchi H, Hayakawa T (2004). Targeted adenovirus vectors.. Hum Gene Ther.

[38] Furue MK, Na J, Jackson JP, Okamoto T, Jones M (2008). Heparin promotes the growth of human embryonic stem cells in a defined serum-free medium.. Proc Natl Acad Sci U S A.

[39] Makino H, Toyoda M, Matsumoto K, Saito H, Nishino K (2009). Mesenchymal to embryonic incomplete transition of human cells by chimeric OCT4/3 (POU5F1) with physiological co-activator EWS.. Exp Cell Res.

[40] Nagata S, Toyoda M, Yamaguchi S, Hirano K, Makino H (2009). Efficient reprogramming of human and mouse primary extra-embryonic cells to pluripotent stem cells.. Genes Cells.

[41] Mizuguchi H, Kay MA (1998). Efficient construction of a recombinant adenovirus vector by an improved in vitro ligation method.. Hum Gene Ther.

[42] Mizuguchi H, Kay MA (1999). A simple method for constructing E1- and E1/E4-deleted recombinant adenoviral vectors.. Hum Gene Ther.

[43] Tashiro K, Kawabata K, Sakurai H, Kurachi S, Sakurai F (2008). Efficient adenovirus vector-mediated PPAR gamma gene transfer into mouse embryoid bodies promotes adipocyte differentiation.. J Gene Med.

[44] Maizel JV, White DO, Scharff MD (1968). The polypeptides of adenovirus. I. Evidence for multiple protein components in the virion and a comparison of types 2, 7A, and 12.. Virology.

[45] Wilkinson DG, Bhatt S, Herrmann BG (1990). Expression pattern of the mouse T gene and its role in mesoderm formation.. Nature.

