# Supplementary material for: Efficient and Directive Generation of Two Distinct Endoderm Lineages from Human ESCs and iPSCs by Differentiation Stage-Specific SOX17 Transduction
Source: PLoS One. 2011 Jul 7;6(7):e21780. doi: 10.1371/journal.pone.0021780 (PMC3131299; doi:10.1371/journal.pone.0021780)
Supplement: Table S1 — List of Taqman probes and primers used in this study. (DOC) [file pone.0021780.s001.doc]

| Genes | Assay ID or Primers (forward/reverse; 5' to 3') |
| --- | --- |
| GAPDH | Hs99999905_m1 |
| NANOG | Hs02387400_ｇ1 |
| FOXA2 | Hs00232764_m1 |
| SOX17 | Hs00751752_s1 |
| GATA4 | Hs00171403_m1 |
| GSC | Hs00418279_m1 |
| HEX | Hs00242160_m1 |
| SOX7 | Hs00846731_s1 |
| FLK1 | Hs00176676_m1 |
| ALB | Hs00910225_m1 |
| CYP2D6 | Hs02576168_g1 |
| CYP3A4 | Hs00430021_m1 |
| AFP | Hs01040607_m1 |
| CYP7A1 | Hs00167982_m1 |
| GAPDH | GGTGGTCTCCTCTGACTTCAACA/GTGGTCGTTGAGGGCAATG |
| LAMB1 | AGGAACCCGAGTTCAGCTACG/CACGTCGAGGTCACCGAAA |
| hCGα | GTTTCTGCATGTTCTCCATTC/GTGGACTCTGAGGTGACGT |
| hCGβ | TCACCGTCAACACCACCATC/AGAGTGCACATTGACAGCTG |
| GATA2 | ACTCCTTCACTCTCAGAGGC/TCGAGGTGATTGAAGAAGAC |
| CDX2 | CTGGAGCTGGAGAAGGAGTTTC/ATTTTAACCTGCCTCTCAGAGAGC |
