# Supplementary material for: Efficient and Directive Generation of Two Distinct Endoderm Lineages from Human ESCs and iPSCs by Differentiation Stage-Specific SOX17 Transduction
Source: PLoS One. 2011 Jul 7;6(7):e21780. doi: 10.1371/journal.pone.0021780 (PMC3131299; doi:10.1371/journal.pone.0021780)
Supplement: Table S2 — List of antibodies used in this study. (DOC) [file pone.0021780.s002.doc]

| Antigen | Type | Company |
| --- | --- | --- |
| FOXA2 | goat | R&D Systems |
| SOX17 | mouse | R&D Systems |
| NANOG | rabbit | ReproCell |
| T | goat | R&D Systems |
| CXCR4 | mouse | R&D Systems |
| c-Kit | mouse | BD Pharmingen |
| HEX | goat | Santa Cruz Biotechnology |
| SOX7 | goat | R&D Systems |
| ALB | rabbit | SIGMA |
| CK18 | mouse | Invitrogen |
| CYP2D6 | goat | Santa Cruz Biotechnology |
| CYP3A4 | goat | Santa Cruz Biotechnology |
| CYP7A1 | goat | Santa Cruz Biotechnology |
| COUP-TF1 | mouse | Santa Cruz Biotechnology |
| GSC | mouse | Abcam |
