# Supplementary figures and images for: Efficient and Directive Generation of Two Distinct Endoderm Lineages from Human ESCs and iPSCs by Differentiation Stage-Specific SOX17 Transduction
Source: PLoS One. 2011 Jul 7;6(7):e21780. doi: 10.1371/journal.pone.0021780 (PMC3131299; doi:10.1371/journal.pone.0021780)

Supplemental figure 1

A

|                                 |
|---------------------------------|
| medium : MEF conditional medium |
| growth factor : BMP4 (20 ng/ml) |

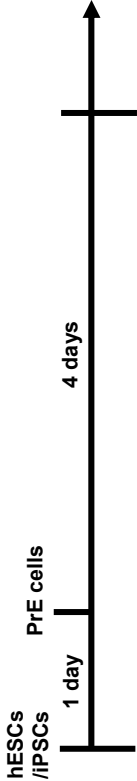

B

C

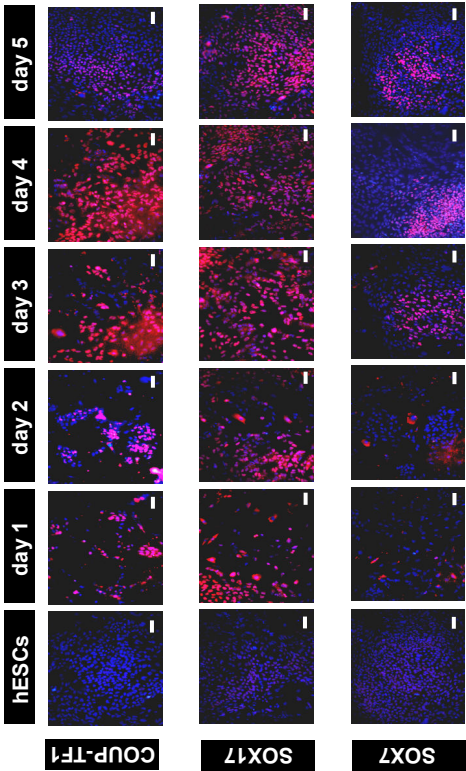

D

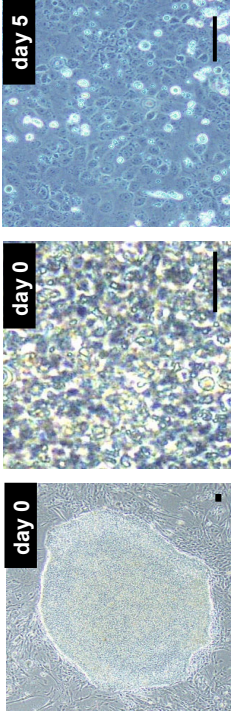

E

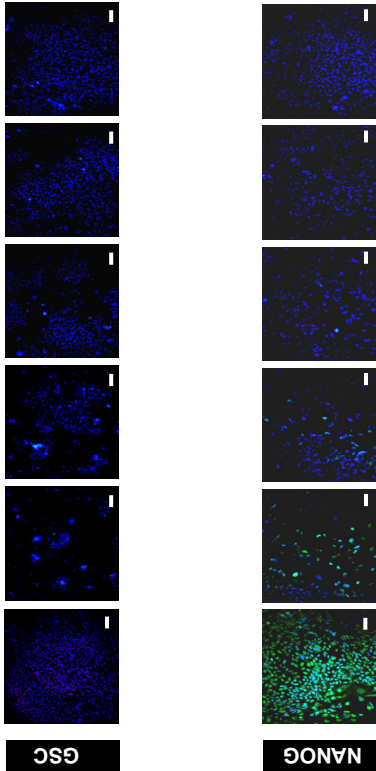

Supplement: Figure S1 — PrE cells formation from human ESCs on day 1 of differentiation. (A) The procedure for differentiation of human ESCs and iPSCs to ExEn cells by treatment with BMP4 (20 ng/ml) is presented schematically. (B) Human ESCs (H9) were morphologically changed during ExEn differentiation; when human ESCs were cultured with the medium containing BMP4 (20 ng/ml) for 5 days, the cells began to show flattened epithelial morphology. The scale bar represents 50 µm. (C–E) The tTemporal protein expression analysis during ExEn differentiation was performed by immunohistochemistry. The PrE markers COUP-TF1 [21] (red), SOX17 [14] (red), and SOX7 [14] (red) were detected on day 1. In contrast to the PS markers, the expression of the DE marker GSC [22] (red) was not detected and the level of the pluripotent marker NANOG (green) declined between day 0 and day 1. Nuclei were counterstained with DAPI (blue). The scale bar represents 50 µm. (PDF) [file pone.0021780.s003.pdf]

Supplemental figure 2

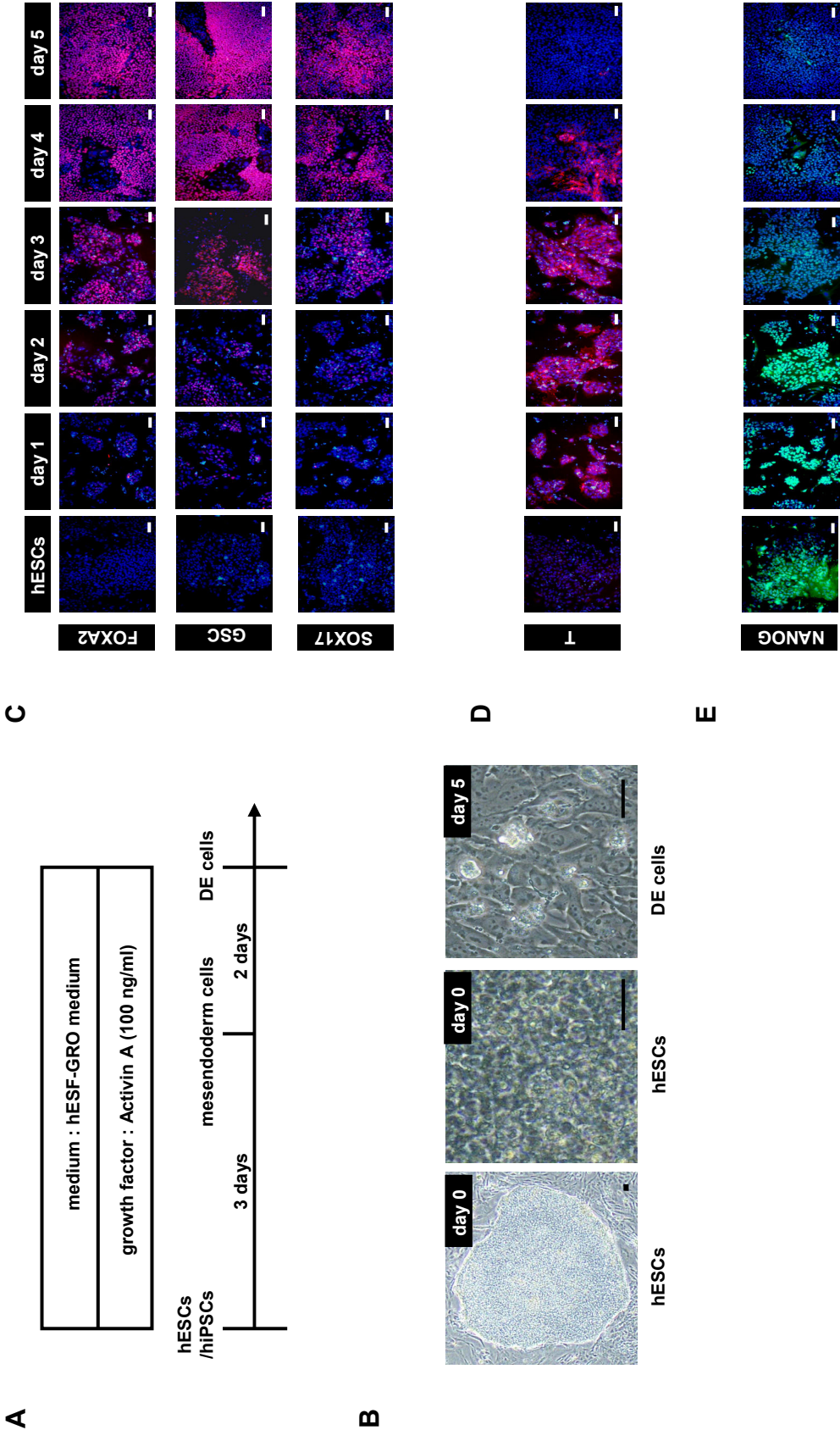

Supplement: Figure S2 — Mesendoderm cells formation from human ESCs on day 3 of differentiation. (A) The procedure for differentiation of human ESCs and iPSCs to DE cells by treatment with Activin A (100 ng/ml) is presented schematically. hESF-GRO medium was supplemented with 5 factors and 0.5 mg/ml fatty acid free BSA, as described in the Materials and Methods. (B) Human ESCs (H9) were morphologically changed during DE differentiation; when human ESCs were cultured with the medium containing Activin A (100 ng/ml) for 5 days, the morphology of the cells began to show visible cell-cell boundaries. The scale bar represents 50 µm. (C–E) The tTemporal protein expression analysis during DE differentiation was performed by immunohistochemistry. The anterior PS markers FOXA2 [21] (red), GSC [22] (red), and SOX17 [14] (red) were adequately detected on day 3. The PS marker T [45] (red) was detected until day 3. In contrast to the PS markers, the expression of the pluripotent marker NANOG [24] (green) declined between day 2 and day 3. Nuclei were counterstained with DAPI (blue). The scale bar represents 50 µm. (PDF) [file pone.0021780.s004.pdf]

Supplemental figure 3

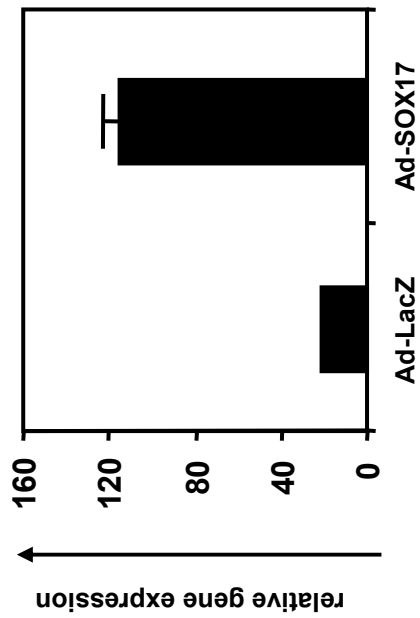

Supplement: Figure S3 — Overexpression of SOX17 mRNA in human ESC (H9)-derived PS cells by Ad-SOX17 transduction. Human ESC-derived PS cells (day 1) were transduced with 3,000VP/cell of Ad-SOX17 for 1.5 h. On day 3 of differentiation, real-time RT-PCR analysis of the SOX17 expression was performed in Ad-LacZ-transduced cells and Ad-SOX17-transduced cells. On the y axis, the expression levels of undifferentiated human ESCs on day 0 was were taken defined as 1.0. All data are represented as the means±SD (n = 3). (PDF) [file pone.0021780.s005.pdf]

Supplemental figure 4

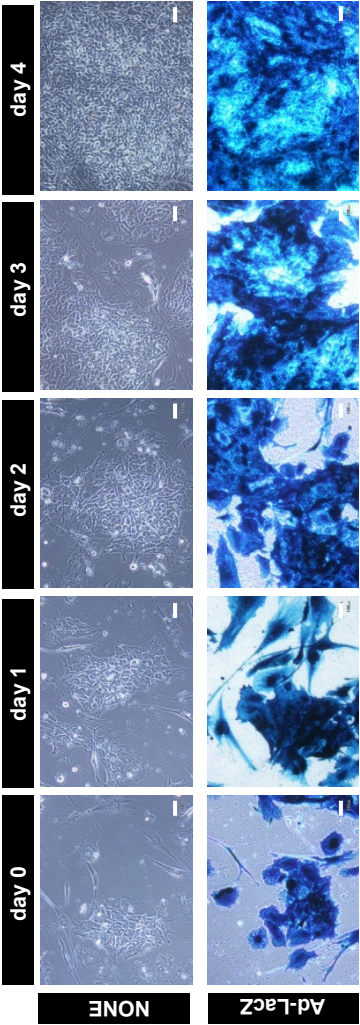

Supplement: Figure S4 — Efficient transduction in Activin A-induced human ESC (H9)-derived cells by using a fiber-modified Ad vector containing the EF-1α promoter. Undifferentiated human ESCs and Activin A-induced human ESC-derived cells, which were cultured with the medium containing Activin A (100 ng/ml) for 0, 1, 2, 3, and 4 days, were transduced with 3,000 vector particles (VP)/cell of Ad-LacZ for 1.5 h. The day after transduction, X-gal staining was performed. The scale bar represents 100 µm. Similar results were obtained in two independent experiments. (PDF) [file pone.0021780.s006.pdf]

Supplemental figure 5

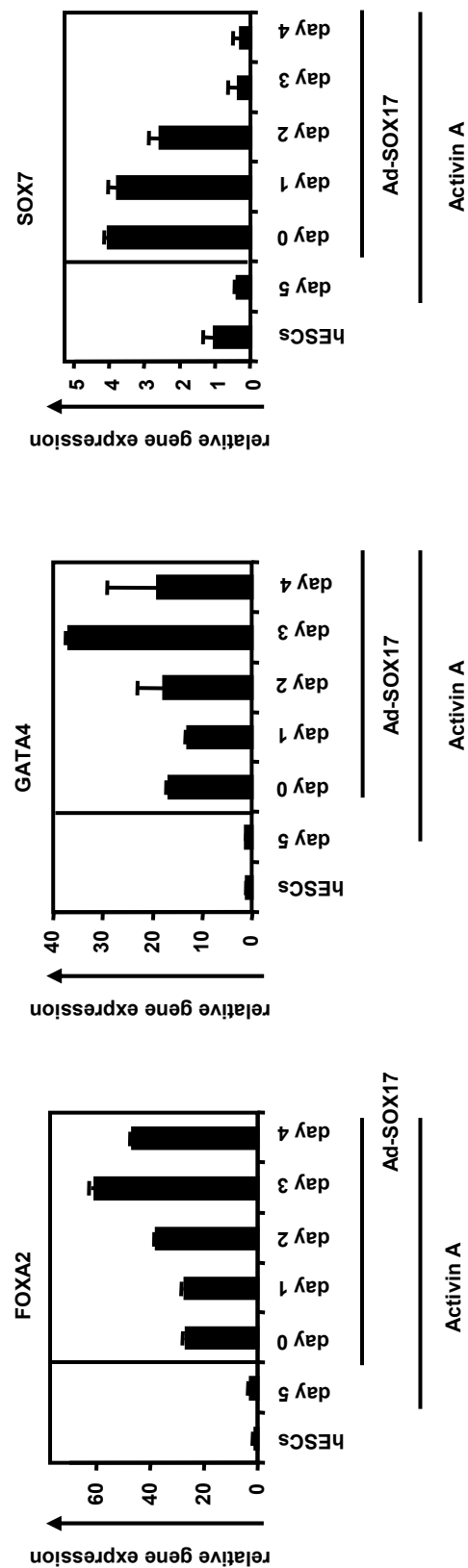

Supplement: Figure S5 — Optimization of the time period for Ad-SOX17 transduction to promote DE differentiation from human iPSCs (Tic). Undifferentiated human iPSCs and Activin A-induced human iPSC-derived cells, which were cultured with the medium containing Activin A (100 ng/ml) for 0, 1, 2, 3, and 4 days, were transduced with 3,000 VP/cell of Ad-SOX17 for 1.5 h. Ad-SOX17-transduced cells were cultured with Activin A (100 ng/ml) until day 5, and then real-time RT-PCR analysis was performed. The horizontal axis represents the day on which the cells were transduced with Ad-SOX17. On the y axis, the expression levels of undifferentiated cells on day 0 was were taken defined as 1.0. All data are represented as the means±SD (n = 3). (PDF) [file pone.0021780.s007.pdf]

Supplemental figure 6

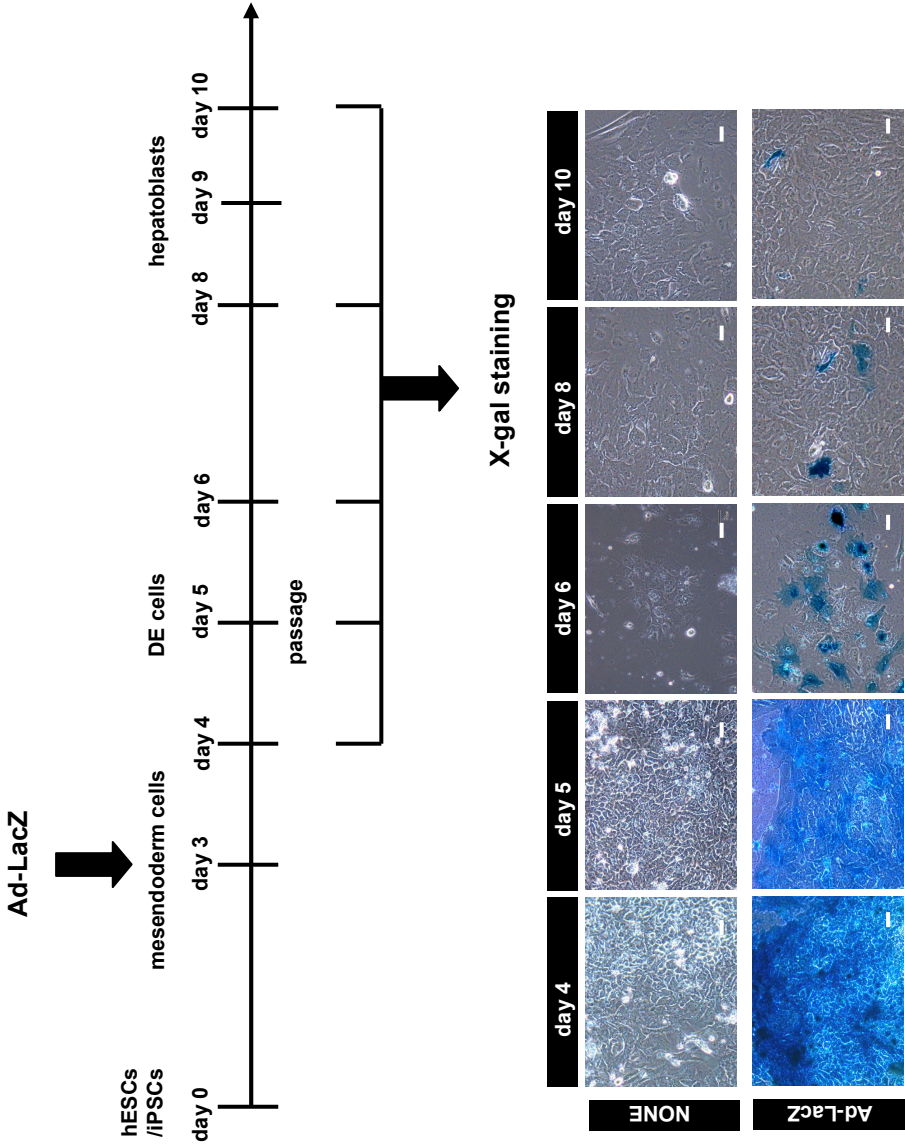

Supplement: Figure S6 — Time course of LacZ expression in human ESC (H9)-derived mesendoderm cells transduced with Ad-LacZ. The hHuman ESC-derived mesendoderm cells (day 3) were transduced with 3,000 VP/cell of Ad-LacZ for 1.5 h. On days 4, 5, 6, 8, and 10, X-gal staining was performed. Note that human ESC-derived cells were passaged on day 5. The scale bar represents 100 µm. Similar results were obtained in two independent experiments. (PDF) [file pone.0021780.s008.pdf]

Supplemental figure 7

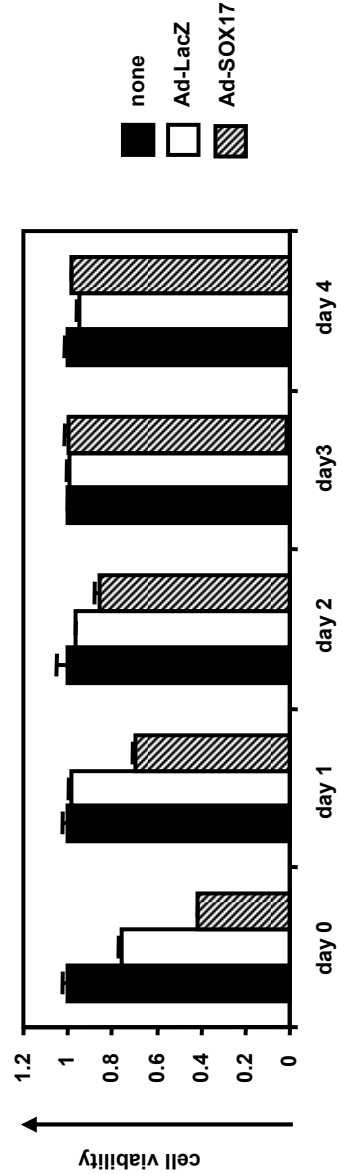

Supplement: Figure S7 — Optimization of the time period for Ad-SOX17 transduction into Activin A-induced human ESC (H9)-derived cells. Undifferentiated human ESCs and Activin A-induced hESC-derived cells, which were cultured with the medium containing Activin A (100 ng/ml) for 0, 1, 2, 3, and 4 days, were transduced with 3,000 VP/cell of Ad-LacZ or Ad-SOX17 for 1.5 h. Ad-SOX17-transduced cells were cultured with Activin A (100 ng/ml) until day 5, then the cell viability was evaluated with crystal violet staining. The horizontal axis represents the day on which the cells were transduced with Ad-SOX17. On the y axis, the level of non-transduced cells was taken defined as 1.0. All data are represented as the means±SD (n = 3). (PDF) [file pone.0021780.s009.pdf]

Supplemental figure 8

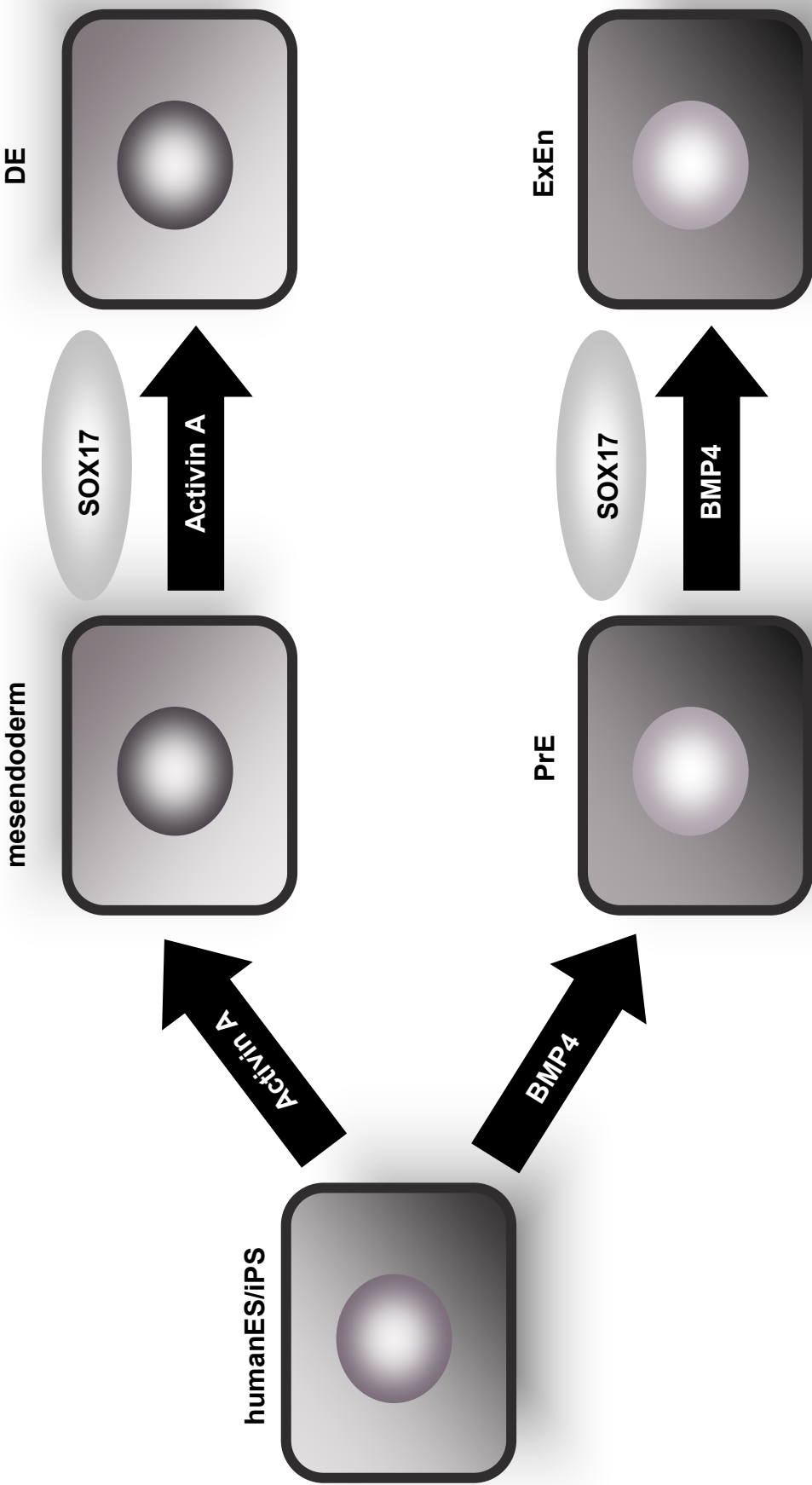

Supplement: Figure S8 — Model of differentiation of human ESCs and iPSCs into ExEn and DE cells by stage-specific SOX17 transduction. The ExEn and DE differentiation process is divided into at least two stages. In the first stage, human ESCs differentiate into either PrE cells by treatment with BMP4 (20 ng/ml) or mesendoderm cells by treatment with Activin A (100 ng/ml). In the second stage, SOX17 promotes the further differentiation of each precursor cell into ExEn and DE cells, respectively. We have demonstrated that the efficient differentiation of these two distinct endoderm lineages is accomplished by stage-specific SOX17 transduction. (PDF) [file pone.0021780.s010.pdf]
